# Allergy to fruit seeds presenting with anaphylaxis

**DOI:** 10.1186/2045-7022-1-S1-P58

**Published:** 2011-08-12

**Authors:** Paul Turner, Paul Gray, Melanie Wong, Dianne Campbell

**Affiliations:** 1 Children's Hospital at Westmead, Allergy & Immunology, Westmead, NSW, Australia

## 

Allergic reactions to fruits, particularly citrus, are relatively common, often presenting with symptoms of oral allergy syndrome (also known as pollen-food syndrome), with systemic allergic reactions occurring less frequently. Fruit allergy testing, including serum specific immunoglobulin E (IgE) and skin prick testing will identify the allergen in most cases, however these tests commonly use extract from the fruit and may fail to identify reactions to the seed, which may be more severe.

We report three children with anaphylactic reactions to fruit seeds, who were able to tolerate the fruit pulp. Two children experienced anaphylaxis to orange seed, and both had evidence of sensitisation to multiple citrus seeds, peanut and tree nuts. The third child developed anaphylaxis to a commercially-produced baby food containing apple puree, and was found to be sensitised to a range of fruit and citrus seeds, as well as to sesame and nuts. These cases highlight the need to consider fruit seeds as a potential cause of severe allergic reactions to fruit.

